# Sequence Variants of Peroxisome Proliferator-Activated Receptor-Gamma Gene and the Clinical Courses of Patients with End-Stage Renal Disease

**DOI:** 10.1155/2015/763459

**Published:** 2015-02-15

**Authors:** Chia-Ter Chao, Yen-Ching Chen, Chih-Kang Chiang, Jenq-Wen Huang, Fu-Chang Hu, Cheng-Chung Fang, Chen-Chih Chang, Chung-Jen Yen

**Affiliations:** ^1^Department of Internal Medicine, National Taiwan University Hospital, Jinshan branch, No. 51, Nanshih, Jinshan district, New Taipei 20844, Taiwan; ^2^Graduate Institute of Toxicology, National Taiwan University, No. 1, Jen-Ai Road Section 1, Taipei 10051, Taiwan; ^3^Institute of Epidemiology and Preventive Medicine, College of Public Health, National Taiwan University, No. 17, Hsu-Chow Road, Taipei 10055, Taiwan; ^4^Department of Internal Medicine, National Taiwan University Hospital, No. 7, Chung-Shan South Road, Taipei 10002, Taiwan; ^5^Department of Integrated Diagnostics & Therapeutics, National Taiwan University Hospital, No. 7, Chung-Shan South Road, Taipei 10002, Taiwan; ^6^Graduate Institute of Clinical Medicine, National Taiwan University, No. 7, Chung-Shan South Road, Taipei 10002, Taiwan; ^7^Department of Emergency Medicine, National Taiwan University Hospital, College of Medicine, National Taiwan University, No. 7, Chung-Shan South Road, Taipei 10002, Taiwan; ^8^Department of Geriatric Medicine and Gerontology, National Taiwan University Hospital, College of Medicine, National Taiwan University, No. 7, Chung-Shan South Road, Taipei 10002, Taiwan

## Abstract

*Background.* PPAR-*γ* single nucleotide polymorphisms (SNPs) reportedly play an important role in determining metabolic risk among diverse population. Whether PPAR-*γ* SNPs affect the clinical courses in ESRD patients is unknown.* Methods.* From a multicenter cohort, we identified 698 patients with prevalent ESRD between 2002 and 2003, and other 782 healthy subjects as control. Two PPAR-*γ* SNPs, Pro12Ala (rs1801282) and C161T (rs3856806), were genotyped and their association with ESRD was examined. Both groups were prospectively followed until 2007, and the predictability of genotypes for the long-term survival of ESRD patients was analyzed.* Results.* After multivariable-adjusted regression, GG genotype of Pro12Ala was significantly more likely to associate with ESRD (*P* < 0.001) among patients with non-diabetes-related ESRD. Cox's proportional hazard regression showed that both Pro12Ala and C161T polymorphisms were significant predictors of mortality in ESRD patients with DM (Pro12Ala: GG versus other genotypes, hazard ratio [HR] <0.01; *P* < 0.001; for C161T, CC versus TT genotypes, HR 2.86; *P* < 0.001; CT versus TT genotypes, HR 1.93; *P* < 0.001).* Conclusion.* This is the first and largest study to evaluate PPAR-*γ* SNPs in ESRD patients. Further mechanistic study is needed to elucidate the role of PPAR-*γ* among ESRD patients.

## 1. Introduction

Chronic kidney disease (CKD) and end-stage renal disease (ESRD) are emerging global epidemics [1–3]. The economic toll brought by complications accompanying CKD and ESRD accounts for over 6% of total medical expenditures in most developed countries [1, 3]. Diabetes mellitus nephropathy (DMN) has been the predominant cause of ESRD worldwide and excellent glycemic control could potentially curb the rise of DM-associated ESRD [4]. Through the amelioration of insulin resistance and hyperinsulinemia, metformin and glitazones are purportedly beneficial for relieving renal injury in DMN [5].

Glitazones are among the most commonly used hypoglycemic agents in patients with DMN. They enhance glucose utilization in skeletal muscles as well as adipokine regulation in adipocytes, among which most effects are mediated by peroxisome proliferator-activated receptors (PPARs), predominantly PPAR-*γ* [6]. PPAR-*γ* is highly expressed in adipocytes and at a much lower level in brain, skeletal muscle, and liver. Owing to the wide distribution, PPAR-*γ* are the masterminds of carbohydrate and lipid metabolism, affect insulin sensitivity, and play a key role in metabolic diseases [7, 8]. Moreover, PPAR-*γ* activation is associated with anti-inflammatory and antiatherogenic effects [9, 10].

Judging from the efficacy of glitazones against DMN and the proinflammatory milieu brought by CKD, PPAR-*γ* could contribute to CKD development and progression [11, 12]. Indeed, sequence variants of PPAR-*γ* correlated with risk of albuminuria and overt DMN [13, 14]. PPAR-*γ* have four mRNA isoforms (PPAR-*γ* 1 to PPAR-*γ* 4) generated by alternative splicing with common sequences encoded by exon 1–6. PPAR-*γ* 2 contains an additional 84 nucleotides (encoded by exon B) at its N-terminus, where clinically important single nucleotide polymorphisms (SNPs) are found [15]. A C-to-G substitution at nucleotide 34 of exon B results in Pro12Ala polymorphism, while C161T of exon 6 represents a synonymous polymorphism without amino acid alteration.

Past literature on PPAR-*γ* polymorphism and CKD is mostly centered on diabetic nephropathy. Pro12Ala polymorphism was associated with albuminuria and DMN risk but not ESRD, and such risk elevation existed in Caucasians but not in Asians [16]. However, studies on nephropathies other than DMN are very rare. One report suggested that C161T of PPAR-*γ* might have a protective effect on the progression of IgA nephropathy [9]. The effect of PPAR-*γ* SNPs on the development and outcomes of non-DM nephropathy have not been investigated before. Consequently, the current study aims to evaluate the relationship between the 2 most common PPAR-*γ* SNPs, Pro12Ala and C161T, and their association with ESRD among patients from Taiwan, where ESRD prevalence is high. Furthermore, we also investigated the overall mortality rate among these ESRD patients according to PPAR-*γ* genetic polymorphisms.

## 2. Material and Methods

### 2.1. Study Population

In this study, ESRD patients (*n* = 698) who underwent maintenance hemodialysis or peritoneal dialysis were recruited from hospitals in northern Taiwan between 2003 and 2004. Individuals who received health check-up (*n* = 782) during the same period of time were also recruited. Blood samples were collected from each subject at the time of enrollment. Both ESRD patients and healthy individuals were followed up prospectively, and survival status was ascertained by the National Death Registry from Taiwan Department of Health between 2003 and 2008. This study was approved by the Institutional Review Boards of National Taiwan University Hospital and its affiliated facilities and adhered to the Declaration of Helsinki. All participants provided written informed consent.

All participants completed a questionnaire about their sociodemographic status (birthday, sex, and education), medical histories (including hypertension and diabetes), and lifestyle (including smoking and alcohol consumption) with the assistance of general practitioners. The diagnosis of DM was made according to a past history of physician-diagnosed DM or two recordings of fasting blood glucose levels ≥ 126 mg/dL (7.0 mmol/L). The causes of ESRD and the date of long-term dialysis initiation were recorded for each ESRD case. The diagnosis of DMN was achieved if they had a DM history of at least 5-year duration before starting maintenance dialysis, presence of diabetic retinopathy, and/or moderate amount of proteinuria (≥100 mg/dL by urinalysis) without active sediments.

### 2.2. Laboratory Assays

Genomic DNA was extracted from peripheral blood leukocytes using the Chemagic DNA Blood kits (Chemagen AG, Baesweiler, Germany). Genotypes of the two SNPs (Pro12Ala and C161T) were determined by the polymerase chain reaction-restriction fragment length polymorphism (PCR-RFLP), as previously described [17]. Briefly, a 270-base-pair (bp) fragment encompassing Pro12Ala was generated from genomic DNA by PCR using 2 primers, which introduces a BstU-I restriction site (CG/CG) only when the C to G substitution at the nucleotide 34 is present. The PCR products were digested with BstU-I (New England BioLab, Beverly, MA, USA), electrophoresed on a 2.5% agarose gel, and stained with ethidium bromide. For Pro12Ala, fragments of 227 bp and 43 bp were observed for GG genotype, fragments of 270 bp, 227 bp, and 43 bp for CG genotype, and a single 270 bp fragment for CC genotype.

For C161T, PCR-RFLP using 2 primers and the restriction enzyme Nla III (New England BioLab, Beverly, MA, USA) were utilized for genotyping. Two fragments, 245 bp and 101 bp, were observed for CC genotype, 245 bp, 204 bp, 101 bp, and 41 bp for CT genotype, and 204 bp, 101 bp, and 41 bp for TT genotype.

### 2.3. Statistical Analysis

Data analysis was performed using the SAS, 9.1.3 software (SAS Institute Inc., Cary, NC, USA). Two-sided *P* value ≤ 0.05 was considered statistically significant. The potential risk factors and the prognostic factors for these ESRD patients were examined in univariate analysis using chi-square test and Fisher's exact test (categorical), Student's *t*-test (continuous), and Mann-Whitney *U* test (nonparametric), as appropriate. Next, multivariable analysis was conducted using logistic regression model (for factors associated with ESRD presence) and Cox's proportional hazards model (for factors predictive of mortality) to estimate the effects of risk factors on the probability of ESRD and the prognostic factors for long-term mortality among the ESRD patients.

To ensure the quality of analysis results, basic model-fitting techniques for (1) variable selection, (2) goodness-of-fit (GOF) assessment, and (3) regression diagnostics were used in our regression analyses. Specifically, the stepwise variable selection procedure was applied to obtain the candidate final regression model. All the univariate significant and nonsignificant relevant covariates (demographic profiles, comorbidities, etiology of ESRD, creatinine, and genotyping results) and their interaction terms were selected and the significance levels for entry (SLE) and for stay (SLS) were set between 0.15 and 0.35. Both the GOF measures (estimated area under receiver operating characteristic [AUROC] curve and adjusted generalized *R* ^2^) and the GOF tests (Hosmer-Lemeshow test for logistic regression model and Grønnesby-Borgan test for Cox's proportional hazards model) were examined.

## 3. Results

A total of 698 prevalent ESRD patients and 782 healthy individuals were enrolled in this study. The average age of ESRD patients was 58.8 years, with a dialysis vintage of 5.4 ± 4.5 years. The etiologies of ESRD were as follows: chronic glomerulonephritis (33.5%), DM nephropathy (24.6%), polycystic kidney diseases (2%), and others (39.9%). Healthy participants showed no deviation from the Hardy-Weinberg equilibrium (HWE) (Table 1). The deviation from HWE among cases for PPAR-*γ* Pro12Ala (*P* = 0.006) indicated possible association between this SNP and the presence of ESRD.

The genotype distributions and the other clinical characteristics were compared between ESRD and healthy participants (controls) in Tables 2 and 3. Notably, the mean level of serum creatinine (mg/dL) was significantly higher in ESRD group (ESRD versus controls: 11.2 versus 1.0; *P* < 0.001), while serum albumin (g/dL) was higher in controls (ESRD versus controls: 3.9 versus 4.4; *P* < 0.001). The proportion of patients with DM was also higher in cases (ESRD versus controls: 37.7% versus 10.6%; *P* < 0.001). The distribution of the PPAR-*γ* Pro12Ala genotype contained significant differences between the cases and controls, with ESRD groups having significantly more CG genotype and less CC genotype. PPAR-*γ* C161T genotype frequencies did not differ between groups (*P* = 0.482). In addition, a subanalysis according to patients with non-DM or DM related ESRD found that those with DM-related ESRD were significantly more likely to have PPAR-*γ* Pro12Ala CG genotypes but less CC genotypes (*P* = 0.027) (Table 3).

We next attempted to determine the factors associated with the presence of ESRD, according to their PPAR-*γ* SNP statuses. As shown in Table 4, multivariable logistic regression analysis identified three significant predictors for carrying ESRD. Conditioning on age and DM, subjects with non-DM ESRD and GG genotype of PPAR-*γ* Pro12Ala had higher chances to have ESRD (odds ratio [OR] > 1 × 10^6^, *P* < 0.001).

We further assessed the predictability of long-term survivals for the ESRD patients, by genotypes of PPAR-*γ* SNPs. The distributions of factors associated with mortality were compared between survivors and those who died among ESRD patients (Table 5). The mean age was significantly higher in ESRD patients who died (survivors, 54.7 ± 13.4 versus nonsurvivors, 67.8 ± 13.1; *P* < 0.001), with a higher proportion of DM among nonsurvivors (survivors, 29.3% versus nonsurvivors, 56.2%; *P* < 0.001). Survivors had significantly higher levels of serum albumin (*P* < 0.001) and hemoglobin (*P* = 0.001). No significant heterogeneity was found for the distributions of PPAR-*γ* Pro12Ala and PPAR-*γ* C161T genotypes between survivors and nonsurvivors. Cox's proportional hazards model identified four important predictors for mortality (age, PPAR-*γ* Pro12Ala-GG, DM × C161T-CC, and DM × C161T-CT) in ESRD patients (Table 6). Conditioning on age, ESRD patients with GG genotype of PPAR-*γ* Pro12Ala had the lowest risk of mortality during follow-up (hazard ratio [HR] < 0.001; *P* < 0.001) compared with those with the other genotypes. ESRD patients with DM and PPAR-*γ* C161T CT genotype and CC genotype also had significantly higher risk of mortality (the former, HR 1.93, 95% confidence interval [CI] 1.36–2.73; *P* < 0.001; the latter, HR 2.86, 95% CI 2.11–3.86; *P* < 0.001) than those with TT genotype.

## 4. Discussion

To the best of our knowledge, this study showed the first time that PPAR-*γ* SNPs play an important role in both the development of ESRD and the outcomes of these ESRD patients, among a large group of Han Chinese participants. Specifically, ESRD patients were more likely to have PPAR-*γ* Pro12Ala GG genotype than other genotypes. However, for ESRD patients of Han origin, PPAR-*γ* Pro12Ala and C161T demonstrated significant influence on the outcomes among those with concurrent DM, while only Pro12Ala affected the outcomes of ESRD patients without DM.

Common SNPs of PPAR-*γ* have been reported to correlate with elevated risk of DM, coronary artery diseases (CAD), metabolic syndromes, and fatty liver diseases [18–20]. However, the inherent risk differs between ethnicities. A meta-analysis found that CC genotype (Pro/Pro) of PPAR-*γ* Pro12Ala was associated with lower risk of polycystic ovarian syndrome in Europeans but not in Asians [21]. Similarly, European Caucasians with CC genotype of Pro12Ala had lower risk of Crohn's disease than those with GG genotype, not in Asians [22]. Meanwhile, the PPAR-*γ* C161T polymorphism also plays an important role in determining the metabolic risk of Asians. CC genotype of C161T predisposed the young Japanese to the development of metabolic syndrome [23]. C161T-T allele carriers also displayed a 26% risk reduction compared with other genotypes in the Chinese but not in Caucasians [24]. Collectively, these data suggested the context- and ethnicity-dependent risk determination of different PPAR-*γ* SNPs. In addition, even among the so-called Asian population, there might be inherent differences in SNPs distributions and physiologic importance [25].

For patients with nephropathy, the importance of PPAR-*γ* SNPs cannot be overstated. A meta-analysis found that CC genotype of Pro12Ala demonstrably increased risk of DMN among type 2 DM patients compared with other genotypes [16]. For patients with type 1 DM/DMN, GG genotype of Pro12Ala conferred a higher risk of ESRD progression and mortality [26]. However, this risk elevation for DMN was found to be present in Caucasians only [16]. A paucity of studies existed concerning the role of PPAR-*γ* SNPs in patients with non-DM nephropathy, and none were performed in Asians. Yao et al. discovered that CC genotype carriers of both Pro12Ala and C161T had significantly higher serum inflammatory markers, and CC genotype of Pro12Ala (Pro/Pro) predisposed ESRD patients to higher mortality [27]. In the current study, we identified that GG genotype of Pro12Ala, but not C161T, was more likely to be associated with ESRD in nondiabetic patients of Han ethnicity and portended a significantly lower risk of mortality for patients with ESRD, concordant with Yao's finding [27]. While C161T CC genotype did not have survival advantage in ESRD Caucasians, CC genotype carriers of Han ethnicity with DM had poorer survival than the other C161T genotypes (Table 6).

The differential influence of PPAR-*γ* SNPs on the development of ESRD between difference ethnicities is interesting. We proposed several explanations for the higher likelihood of ESRD associated with GG genotype of Pro12Ala in our cohort. First, as explained above, ethnic differences might be an important determinant of the biologic phenotypes of SNPs. CC genotype of Pro12Ala might play a protective role against ESRD development while being detrimental in Caucasians. Pro12Ala GG and CG genotypes have each been found to be associated with obesity development in Asians, compared with CC genotype [19, 28], and obesity is an important predisposing factor for ESRD [29, 30]. Second, this higher likelihood of ESRD in GG genotype carriers might represent the premature mortality of the other patients, precluding their enrollment. Indeed, one report suggested that CC genotype of Pro12Ala elevates the risk of CAD in the Han Chinese, indicating the potentially negative health effect for carriers [20]. Still other studies showed that carrying C allele of Pro12Ala is linked with higher risk of developing colorectal cancers in Asians, another cause of premature mortality before reaching ESRD [31]. Finally, the rarer GG genotype of Pro12Ala has been found to predict type 2 DM development in Asians [32], and the percentage of GG genotype carriers in our cohort was very low. Then the higher risk of ESRD by Pro12Ala GG genotype might reflect partially the higher risk of DM and the subsequent ESRD associated with Pro12Ala GG genotype.

Yao et al. had shown that higher inflammation status could explain the higher mortality introduced by PPAR-*γ* Pro12Ala CC genotype [27]. Although we also identified CC genotype as risk factors for mortality in ESRD patients of Han origin, the degree of risk elevation seems to be higher (Caucasians versus Asians, 2 versus 3) (Table 6). Past reports indicated that Asians had peculiar gene signatures predisposing them to persistent inflammation and a tendency for cardiovascular events and cancers, not found in Caucasians [33, 34]. Thus, the higher risk introduced by PPAR-*γ* Pro12Ala CC genotype might reflect a synergistic effect between multiple Asian-specific SNPs of genes involved in inflammation pathways.

In addition, we also identified PPAR-*γ* C161T C allele carriers to be a mortality predictor of ESRD patients of Han origin (Table 6), compatible with studies describing that PPAR-*γ* C161T polymorphism is associated with risk of CAD in the Chinese only, not among Caucasians [24]. Another Hong Kong study also showed that T allele at the 161 position predicts higher risk of cardiovascular and all-cause mortality in patients with DMN [35]. The synonymous output of C161T SNPs could represent linkage disequilibrium and a potential association with other adjacent gene signatures vital for patient survivals. Consequently, we proposed that PPAR-*γ* C161T polymorphism represents a novel prognostic marker for ESRD patients of Han ethnicity with DM.

Our study has its strengths and limitations. This is the first focusing on the influences of PPAR-*γ* SNPs among ESRD patients of Asian origin, and its credibility is greatly enhanced by the large sample size. However, several limitations did exist. First, only two SNPs were investigated, and a systematic approach, for example, selection of haplotype-tagging SNPs, may enable better visualization of the whole picture. Second, the minor allele frequency of the exon B in ESRD patients failed to satisfy the Hardy-Weinberg equilibrium. This finding might be partially due to the protective effect exerted by CC genotype against ESRD development. Further prospective studies are needed to affirm our findings.

## 5. Conclusion

Our results extend the previous findings of PPAR-*γ* SNPs phenotypic influences, affirming their role in the development of ESRD and prognosis of ESRD in patients of Han origin. More importantly, we discover the C161T polymorphism to be a unique prognostic predictor among ESRD patients, not reported before. Mechanistic studies are needed to investigate the associations between PPAR-*γ* SNPs Pro12Ala, C161T, and their clinical manifestations.

## Figures and Tables

**Table 1:** Minor allele frequency and Hardy-Weinberg equilibrium of two PPAR-*γ* SNPs.

SNP name	rs #	Nucleotide change (amino acid change)	Location	Controls		Cases	
				MAF (%)	HWE *P* value	MAF (%)	HWE *P* value
Pro12Ala	rs1801282	C → G (Proline → Alanine)	Exon B	3.07	0.752	4.87	0.006
C161T	rs3856806	C → T (Histidine → Histidine)	Exon 6	24.23	0.688	26.15	0.520

MAF: minor allele frequency; HWE: Hardy-Weinberg equilibrium.

**Table 2:** Characteristics of the study population by case and control status.

Variables	Controls	Cases	*P* ^*^
Number of subjects	782	698	
Age (years)	60.0 ± 19.2	58.8 ± 14.6	<0.001
Male	412 (52.2%)	334 (44.8%)	0.068
DM	83 (10.6%)	263 (37.7%)	<0.001
Serum creatinine (mg/dL)	1.0 ± 0.3	11.2 ± 2.5	<0.001
Serum albumin (g/dL)	4.4 ± 0.2	3.9 ± 0.5	<0.001
PPAR-*γ* Pro12Ala genotype			0.037
CC	735 (94.0%)	635 (91.1%)	
CG	46 (5.9%)	58 (8.2%)	
GG	1 (0.1%)	5 (0.7%)	
PPAR-*γ* C161T genotype			0.482
CC	451 (57.7%)	384 (55.0%)	
CT	283 (36.2%)	263 (37.7%)	
TT	48 (6.1%)	51 (7.3%)	
Death	39 (5.0%)	217 (31.1%)	<0.001

^*^Continuous variables were tested by Mann-Whitney *U* tests, whereas categorical variables were tested by Fisher's exact test.

**Table 3:** Genotype distributions of the study population by cases with DM, cases without DM, and control status.

Variables	Controls	Cases_non-DM	Cases_DM	*P* ^*^
Number of subjects	782	512	186	
PPAR-*γ* Pro12Ala genotype				0.027
CC	735 (94.0%)	471 (92.0%)	164 (88.2%)	
CG	46 (5.9%)	37 (7.2%)	21 (11.3%)	
GG	1 (0.1%)	4 (0.8%)	1 (0.5%)	
PPAR-*γ* C161T genotype				0.673
CC	451 (57.7%)	278 (54.3%)	106 (57.0%)	
CT	283 (36.2%)	198 (38.7%)	65 (34.9%)	
TT	48 (6.1%)	36 (7.0%)	15 (8.1%)	

^*^Continuous variables were tested by Mann-Whitney *U* tests, whereas categorical variables were tested by chi-square test or Fisher's exact test.

**Table 4:** The predictors of ESRD identified by fitting multiple logistic regression model weighted by the inverse of survival probability using the stepwise variable selection method.

Covariate^*^	Regression coefficient	Standard error	*t* value	*P* value	Odds ratio	95% confidence interval of odds ratio		
Intercept	0.075	0.192	0.390	0.697				
Age (years)	−0.008	0.003	−2.666	0.008	0.992	0.985	—	0.998
DM	1.811	0.141	12.846	<0.001	6.225	4.724	—	8.203
Non-DM × PPAR-*γ* Pro12Ala-GG	13.939	0.511	27.266	<0.001	1.1 × 10^6^	4 × 10^5^	—	3 × 10^6^

Multiple weighted logistic regression model: *n* = 1480; the estimated area under the receiver operating characteristic (ROC) curve = 0.689.

DM: diabetes mellitus; ESRD: end-stage renal disease.

^*^The symbol “×” indicates the interaction between two covariates and it can be literally interpreted as “and” in this case.

**Table 5:** Univariate analysis of risk factors for mortality in dialysis cases.

Variables	Alive (*n* = 481)	Dead (*n* = 217)	*P* ^*^
Age (years)	54.7 ± 13.4	67.8 ± 13.1	<0.001
Male	224 (46.5%)	110 (50.7%)	0.327
DM	141 (29.3%)	122 (56.2%)	<0.001
Serum albumin (g/dL)	4.0 ± 0.4	3.7 ± 0.5	<0.001
Serum hemoglobin (g/dL)	10.2 ± 1.5	9.8 ± 1.7	0.001
PPAR-*γ* Pro12Ala genotype			0.417
CC	435 (90.5%)	200 (92.2%)	
CG	41 (8.5%)	17 (7.8%)	
GG	5 (1.0%)	0 (0%)	
PPAR-*γ* C161T genotype			0.332
CC	257 (53.3%)	127 (58.6%)	
CT	185 (38.5%)	78 (35.9%)	
TT	39 (8.1%)	12 (5.5%)	

^*^Continuous variables were tested by Mann-Whitney *U* tests, whereas categorical variables were tested by Fisher's exact test.

**Table 6:** The predictors of time to death in dialysis cases by fitting multiple Cox's proportional hazards model^**^ weighted by the inverse of survival probability using the stepwise variable selection method.

Covariate^*^	Regression coefficient	Standard error	*z*	*P* value	Hazard ratio	Lower 95% limit	Upper 95% limit
Age (years)	0.065	0.006	10.707	<0.001	1.068	1.055	1.080
PPAR-*γ* Pro12Ala-GG	−14.61	0.695	−21.021	<0.001	<0.01	<0.01	<0.01
DM × PPAR-*γ* C161T-CC	1.050	0.156	6.728	<0.001	2.858	2.105	3.858
DM × PPAR-*γ* C161T-CT	0.655	0.178	3.675	<0.001	1.926	1.358	2.731

^*^The symbol “×” indicates the interaction between two covariates and it can be literally interpreted as “and” in this case.

^**^Weighted Cox's proportional hazards model: *n* = 698, number of events = 217, and concordance = 0.756 (se = 0.021).
